# The Segond Fracture as a Vital Diagnostic Tool for Anterior Cruciate Ligament Injury in Acute Settings: A Case Report

**DOI:** 10.7759/cureus.58588

**Published:** 2024-04-19

**Authors:** Benjamin D Gompels, Ojorhelumi C Kanwei, Stephen McDonnell

**Affiliations:** 1 Department of Surgery, University of Cambridge, Cambridge, GBR; 2 Department of Trauma and Orthopaedics, Addenbrooke's Hospital, Cambridge University Hospitals NHS Foundation Trust, Cambridge, GBR; 3 Department of Trauma and Orthopaedics, University of Cambridge, Cambridge, GBR

**Keywords:** orthopaedic research, orthopaedic emergency, ortho surgery orthopaedics surgery bones joints hip knee shoulder hand foot ankle elbow arthroplasty arthroscopy replacement tendons ligaments prosthesis implants cartilage osteoarthritis arthritis, sports surgery, msk radiology, ortho surgery

## Abstract

A Segond fracture is a specific type of avulsion fracture involving the lateral aspect of the proximal tibia adjacent to the tibial plateau. Segond fractures are indicative of ligamentous injury in the knee. In this case report, a 29-year-old male delivery driver presented to the ED with acute onset right knee pain after losing control of his motorbike at low speed. Examination revealed significant effusion and medial and lateral joint line tenderness. An anterior-posterior radiograph of the knee showed a Segond fracture. Subsequent MRI confirmed a full-thickness anterior cruciate ligament (ACL) rupture and medial collateral ligament (MCL) tear. Despite surgical reconstruction options, the patient chose conservative management. At eight-week follow-up, he demonstrated satisfactory progress. This case highlights the diagnostic significance of Segond fractures in identifying ligamentous damage in the knee without the availability of MRI. It also highlights the feasibility of non-operative management in some instances.

## Introduction

Anterior cruciate ligament (ACL) injuries are a common orthopedic concern, particularly among young, active individuals. According to the UK National Ligament Registry, 18,668 patients presented with an ACL injury in the UK from December 1, 2012, to December 1, 2021, highlighting a significant burden. While ACL injuries can occur across all age groups, they most commonly affect young individuals, often during sports or recreational activities, with a higher incidence in female athletes than in males [1].

In the acute setting, patients with ACL injuries typically present with a history of a traumatic event, such as sudden deceleration, pivoting, or twisting motion of the knee joint, often accompanied by a popping sensation. This acute mechanism of injury is frequently observed during sports activities like football, rugby, or skiing, where rapid changes in direction or collisions can significantly stress the ACL.

The clinical examination of patients with suspected ACL injuries often reveals signs of acute knee instability, including joint effusion, tenderness along the joint line, and pain with weight-bearing and range of motion. Additionally, the Lachman and anterior drawer tests, which assess the anterior translation of the tibia relative to the femur, are commonly performed to evaluate ACL integrity. Positive findings on these tests, coupled with a history suggestive of an ACL injury, warrant further diagnostic evaluation, primarily through imaging studies (i.e., MRI).

## Case presentation

**Figure 1:**
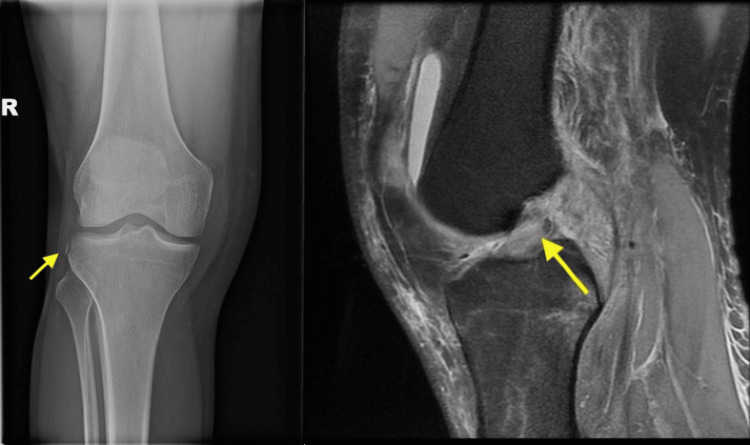
Anteroposterior (AP) radiograph of the lateral tibial plateau (left, panel A), MRI of the knee (right, panel B). A plain anterior-posterior radiograph showed an avulsion fracture of the lateral tibial plateau (Panel A). An MRI, completed as an outpatient, confirmed a full-thickness ACL rupture, and a concurrent MCL rupture was noted on the MRI (Panel B).

A 29-year-old male delivery driver presented to the ED with acute onset right knee pain after losing control of his motorbike at low speed. Examination revealed substantial effusion, accompanied by medial and lateral joint line tenderness. A plain anterior-posterior radiograph showed an avulsion fracture of the lateral tibial plateau (Figure 1). MRI, completed as an outpatient, confirmed a full-thickness ACL rupture, and a concurrent medial collateral ligament (MCL) rupture was noted on the MRI (Figure 1). Consequently, the patient opted for conservative management with physiotherapy instead of operative reconstruction and made satisfactory progress at his most recent follow-up.

## Discussion

A 'Segond' fracture is an avulsion fracture involving the lateral aspect of the proximal tibia, named after the eminent French eighteenth-century surgeon Paul Segond [2]. It can be useful to clinicians in the acute setting, as though it may appear as an inconspicuous fracture, it is pathognomonic for ligamentous damage in the knee. Most commonly, the ACL is damaged, but the MCL can also be involved [3]. Following surgical reconstruction, the Segond fracture can often undergo spontaneous healing, although it is tied to developing a residual bony protrusion from the lateral tibial plateau.

Identifying a Segond fracture is a valuable clinical tool that can expedite the diagnosis of ACL tears in the acute setting, especially when there is no access to MRI imaging to visualize the soft tissue structures in the knee joint. Clinicians should be vigilant in recognizing this plain radiographic finding, as it provides essential diagnostic information that may not be apparent on physical examination alone and does so without MRI imaging. In cases where patients present with acute knee injuries and imaging reveals a Segond fracture, clinicians should have a high index of suspicion for concomitant ACL tears, especially in the absence of significant trauma. Prompt recognition of ACL tears facilitated by a Segond fracture enables clinicians to initiate appropriate management strategies early in the course of injury, including timely referral to orthopaedic specialists for further evaluation and consideration of surgical intervention.

The management of ACL tears varies depending on patient-specific factors such as age, activity level, and occupation. Nonoperative management, including physical therapy, bracing, and activity modification, may be successful in less active patients with sedentary jobs [4]. Patients with minor instability (defined as grade ≤ 1) on the Lachman and pivot-shift tests may achieve reasonable knee function with non-operative treatment, improving acute impairments and regaining muscle strength to minimize the risk of functional instability or episodes of giving way [5]. Rehabilitation alone is recommended before a return to sports, with the completion of a structured program to ensure a safe return and reduce the risk of further knee injuries [5]. However, despite rehabilitation efforts, some patients struggle to regain acceptable knee function and joint stability, which decreases physical activity and quality of life [5]. While the evidence supporting non-operative interventions before surgical intervention for ACL rupture is limited, it suggests that non-operative treatment should be attempted before considering surgery [6]. However, meta-analysis data favor early surgical stabilization over non-operative or delayed treatment, with patients experiencing more instability and an inability to return to previous activity levels with non-operative or delayed treatment [7]. Physiotherapy-led interventions such as Pilates and Tai Chi may improve pain, proprioception, and strength in young and middle-aged adults with partial ACL tears. However, further high-quality randomized studies with long-term outcomes are needed to validate these findings [8]. The decision between non-operative management and surgical intervention for ACL tears should be individualized based on patient preferences, activity level, and response to conservative treatments.

## Conclusions

Identifying a Segond fracture is an important clinical sign, pathognomonic of an ACL injury. Identification in the acute setting on plain films can guide clinicians toward appropriate referral to secondary care without the need for MRI. This can improve patient outcomes through timely consideration of operative or non-operative management.
